# Editorial: The Role of Calcium Handling in Heart Failure and Heart Failure Associated Arrhythmias

**DOI:** 10.3389/fphys.2019.00001

**Published:** 2019-01-22

**Authors:** Daniel M. Johnson, Alessandro Mugelli, Elisabetta Cerbai

**Affiliations:** ^1^Institute of Cardiovascular Sciences, University of Birmingham, Birmingham, United Kingdom; ^2^Department of Neuroscience, Psychology, Drug Research and Child Health (NeuroFarBa), University of Florence, Florence, Italy

**Keywords:** calcium, arrhythmia, heart failure, calcium signaling, ventricular, atrial

Over 20 years ago Heart Failure (HF) was described as an epidemic (Braunwald, 1997) and although we have made substantial progress in HF research and treatment since then, the prevalence of HF continues to rise. The estimated 6.5 million American adults over the age of 20 with HF between 2011 and 2014 (Benjamin Emelia et al., 2018) calls for a deeper understanding of mechanisms involved in this potentially life threatening syndrome.

Ever since Sydney Ringer's seminal work in the late 1800's proving the importance of Ca^2+^ in contractility (Miller, 2004), we have continued to be astounded by this multifaceted performer of cardiac function. In addition to sustaining the normal heartbeat, once dysregulated, Ca^2+^ handling and its associated proteins and pathways may turn into major driving factors toward mechanical and electrical dysfunction in HF.

Over recent years substantial progress has been made in understanding the links between Ca^2+^ handling and HF, including the arrhythmogenic predisposition. For these reasons the present Research Topic focuses on the interactions of Ca^2+^ with multiple players that can influence HF progression and arrhythmia liability. It contains both review articles as well as original research in this area which we believe could contribute to our understanding of HF and potential therapeutic targets.

The topic opens with two overview articles on Ca^2+^ handling and arrhythmogenesis in two relevant pathological conditions (Coppini et al.; Johnson and Antoons). Johnson and Antoons focus on the pathways likely contributing to ventricular arrhythmogenesis in HF with reduced ejection fraction (HFrEF) as a result of changes in both stretch and β-adrenergic signaling. These authors consider the changes in signaling at the level of the single cardiac myocyte in HF, looking at the crosstalk between the two complex signaling pathways associated with HF, Ca^2+^ handling and arrhythmogenesis, their disruption in HF and finally the consequence of changes in local Ca^2+^ microdomains (Johnson and Antoons). Coppini et al. describe changes in intracellular Na^+^ and Ca^2+^ in hypertrophic cardiomyopathy (HCM) using human ventricular tissue and single myocytes. HCM is one of the most common inherited cardiac diseases, generally due to mutations in sarcomeric proteins, and can lead to severe diastolic dysfunction and sudden death (Coppini et al.). In both papers, a number of potential therapeutic targets are proposed.

A link between atrial fibrillation (AF), HF and sudden cardiac death has been apparent for a number of years, (Lee Park and Anter, 2013; Reinier et al., 2014), and two articles aim to shed light into novel links between these “epidemics.” Initially Denham et al. provide an excellent and extensive review of the literature linking AF and HF with altered Ca^2+^ handling, and discuss how these two “syndromes” feedback on each other eventually worsening the phenotype. Then, Molina et al. provide novel insights into this subject using explanted tissue and myocytes isolated from patients with HFrEF alone or HFrEF with concomitant chronic AF. Interestingly, a number of differences were noted between these two populations, including increased spontaneous I_NCX_ and RyR2 open probability in patients with both AF and HFrEF when compared to patients with HFrEF alone (Molina et al.). These observations set a starting point in the “chicken and egg” history of AF, suggesting the occurrence of a specific substrate triggering AF in the context of HF-induced remodeling, that could be translated into improved therapeutic options.

Improvements in imaging techniques have speeded up our understanding of Ca^2+^ handling in multiple organ systems and pathologies, and HF has been no exception. Sacconi's lab has been amongst the pioneers in this area with the development of the ultrafast random access multiphoton (RAMP) microscope. This approach was used to simultaneously measure action potentials (AP) and intracellular Ca^2+^ transients at multiple sites, and in different sub-cellular domains within the myocyte (Crocini et al., 2014). Furthermore, this technique allows investigation of spatio-temporal relationships between local Ca^2+^ release and AP prolongation. Recent findings from this area are described in the current research topic by Scardigli et al. Ghouri et al. also utilize novel imaging techniques to optically image intact (infarcted) hearts. Using 2-photon microscopy, they visualize functional differences below the surface layers thus demonstrating electrophysiological abnormalities in infarcted areas when compared to the border zone and the non-infarcted area (Ghouri et al.).

*In-silico* investigations have also had a large impact on our understanding of cardiac electrophysiology over the years, as they allow dissecting of pathways to a resolution that may not be possible in the “wet-lab” (Quinn and Kohl, 2013). Mora et al. specifically look at the interactions between fibroblasts and myocytes using mathematical modeling and how these interactions can alter Ca^2+^ handling. Meanwhile, Tomek et al. expand our knowledge on how action potential alternans can be regulated by differential Ca^2+^ release from the sarcoplasmic reticulum (Mora et al.; Tomek et al.).

The search for unusual or veiled culprits—such as specific protein interactions or pathways—likely contributing to arrhythmia in HF is the common topic of three papers. Arvanitis et al. give an overview of the Histidine Rich Ca^2+^ binding protein (HRC) and its potential role in arrhythmogenesis. whilst Skogestad and Aronsen unravel alterations as a result of hypokalemia—a common syndrome amongst HF patients with life-threatening pro-arrhythmic consequences. Meanwhile, Ronchi et al. investigate how three factors often altered in HF- namely sympathetic pathways, AP duration and the renin-angiotensin system- can lead to altered Ca^2+^ release from the sarcoplasmic reticulum.

Finally, prediction of potential arrhythmias in HF patients remains challenging. Sprenkeler et al. use a large animal model with ventricular remodeling to demonstrate that differences in force-frequency relationships and mechanical restitution may be an interesting avenue to pursue. These data should ignite studies aimed at investigating the clinical relevance of such parameters (Sprenkeler et al.).

Overall, this issue of Frontiers has highlighted the fact that Ca^2+^ research in HF and the link to arrhythmogenesis is flourishing. There are still many unexplored avenues for research, exploiting state-of-the-art technologies to gain deeper insight into individual or common arrhythmogenic mechanisms in HF with different etiology and dysfunction (e.g., HFrEF vs. HFpEF).

We hope that this Topic will stimulate the development of future collaborative and interdisciplinary research ultimately leading to an increased therapeutic arsenal against life-threatening arrhythmias in HF.

## Author Contributions

All authors listed have made a substantial, direct and intellectual contribution to the work, and approved it for publication.

### Conflict of Interest Statement

The authors declare that the research was conducted in the absence of any commercial or financial relationships that could be construed as a potential conflict of interest.
